# A formative study to inform mHealth based randomized controlled trial intervention to promote exclusive breastfeeding practices in Myanmar: incorporating qualitative study findings

**DOI:** 10.1186/s12911-016-0301-8

**Published:** 2016-06-04

**Authors:** Myat Pan Hmone, Michael J. Dibley, Mu Li, Ashraful Alam

**Affiliations:** Sydney School of Public Health, Sydney Medical School, The University of Sydney, Sydney, NSW 2006 Australia

**Keywords:** Qualitative formative research, mHealth, Text messaging, Pregnant woman, Service providers, Exclusive breastfeeding, Infant feeding practices, Randomized controlled trial intervention, Nutrition, Myanmar

## Abstract

**Background:**

Undernutrition is a major concern for Myanmar children with low exclusive breastfeeding rate (24%). A formative study was conducted to explore the perceptions and practices relating to exclusive breastfeeding, and barriers and facilitators to using mobile communications for exclusive breastfeeding counselling. The results inform the design of a randomized control trial to promote exclusive breastfeeding practices among Myanmar mothers.

**Methods:**

We conducted twenty in-depth interviews with pregnant women and accompanying family members attending an antenatal clinic at the Central Women’s Hospital, Yangon, seven key-informant interviews and one focus group discussion with fifteen service providers such as nurses, doctors, managers and staff from the National Nutrition Centre, Department of Health, United Nations Children’s Fund International and National Non-Government Organizations and Ooredoo, a private mobile company.

**Results:**

Widespread practices of feeding water, honey, infant formula and semi-solid food were reported to be existed in the community before the child reaches four months, mostly influenced by grandmothers from both sides. All couples knew breast milk was good for baby and intended to breastfeed, though limited understanding of the term exclusive breastfeeding was reported. Perception that breast milk alone was not sufficient to provide all nutrients needed for the first six months of baby’s life, mother had insufficient milk supply or breast problems, mother’s back to work and grandmothers’ influence emerged as barriers to breastfeed exclusively for six months. All women knew how to make basic phone calls, majority could read mobile text message in Burmese and possess mobile phones while a few of them shared phones with their husbands. All couples preferred to receive text messages 2–3 times per week in the evening. Institutional staff suggested messages to be simple, easily understandable and culturally appropriate. Perceived barriers included limited mobile network coverage, affordability of mobile handset and phone bills, literacy and community familiarity with text messages. All respondents welcomed the idea of planned intervention.

**Conclusion:**

We incorporated findings to develop messages and determine the modality, inclusion criteria and tailored with gestation and child age, to be delivered in the randomized controlled trial intervention.

## Background

Myanmar has a high under-five mortality rate with 56 deaths per 1,000 live births and 35% of children under-five years are stunted [1, 2]. Malnutrition has been responsible for 60% of the under- five children deaths annually [3] and under-nutrition contributes to 30% of child deaths globally [4]. The ‘Global Strategy for Infant and Young Child Feeding’ recommendations have clearly pinpointed the importance of breastfeeding [3] for child health. Several scientific studies and reports suggest that effective exclusive breastfeeding promotion programs alone can prevent the leading causes of child deaths such as diarrhoea, pneumonia and neonatal sepsis [5–7]. The Lancet 2013 Maternal and Child Nutrition series and a study in Nepal highlighted that effective exclusive breastfeeding (EBF) and early initiation of breastfeeding promotion programs with high coverage could prevent 13% of under-five children death and almost 20% of neonatal deaths [4, 8, 9].

Keeping this in mind, breastfeeding promotion programs emphasizing early initiation and exclusive breastfeeding should be promoted in resource constrained countries and Myanmar is no exception. Although 90% of women of reproductive age in Myanmar in 2010 were aware of the benefits of breast milk for their babies, only 24% of infants younger than 6 months of age were exclusively breastfed. Myanmar has the third lowest exclusive breastfeeding rate in the Asia-Pacific region [10, 11] and mothers often feed water, breast milk substitutes, pre-lacteals and other complementary feedings within a few weeks to 2–4 months after delivery.

Myanmar is now on the verge of a communications revolution as the mobile phone network is rolled out across the country since 2012 and phones prices have plunged from US $1,000 in 2011 to $15 in 2014–15. We, therefore, plan to conduct a randomized controlled trial to evaluate a mobile phone communications intervention to promote exclusive breastfeeding practices among women from Yangon. To aid the design of the trial, we conducted a formative study to explore the perceptions and practices relating to exclusive breastfeeding, and barriers and facilitators to using mobile communications for exclusive breastfeeding counselling.

## Brief description of M528 planned randomized control trial

The planned randomized controlled trial intervention is called ‘M528 intervention’ where ‘M’ stands for the mobile phone and ‘528’ refers to the bonding and love between mother and baby. The term ‘528’ is commonly used in Myanmar regardless of races or religions to indicate selfless love between the two human beings.

M528 is a two-group parallel arm randomized controlled trial with nine months of follow up from the time of recruitment till the child reaches six months of age. We hypothesize that the exclusive breastfeeding rate in the intervention group will double compared with the control group. After incorporating the findings from this formative study into the trial design and SMS text messages contents, the trial will commence. The study will be conducted in the same setting as a formative study setting which the antenatal clinic at the Central Women Hospital, Yangon. A total of 344 pregnant women between 28–34 weeks of gestation will be recruited for a period of 6 weeks and allocated into the intervention and the control group while allocation sequences will be concealed. Breastfeeding promotional health education messages, tailored for the women’s stage of gestation and the child’s age will be sent three times/week to the intervention group. The control group will receive health messages which did not include information about breastfeeding once a week. Recruited women’s delivery status will be tracked through interactive text messages, phone calls and hospital records. The primary outcome is exclusive breastfeeding rate at the sixth month and secondary outcomes are median duration of exclusive breastfeeding and other infant feeding practices. Outcomes will be assessed via monthly follow-up phone calls postnatally at one to six months in both groups. Breastfeeding self-efficiency scale, social desirability scale, text messages delivery success and user experience on receiving text messages will be assessed.

## Methods

### Study site and population

Central Women’s Hospital, Yangon was purposefully selected because it was the largest public hospital providing free women health services and provided a setting where we could recruit women from various walks of life. Secondly, as our intervention uses a mobile phone platform and this setting currently has the best mobile network coverage in Myanmar. The average antenatal care clinics attendance was approximately 2,000 pregnant women per month [12]. During a preliminary site assessment, we observed that the majority of antenatal care clinic attendees were using mobile phones and from the hospital records appeared to live in areas with high mobile network coverage [12].

The study population comprised of selected pregnant women seeking antenatal care at the Central Women’s Hospital, accompanying family members and service providers.

### Sampling

We applied purposive sampling and selected the pregnant women from the antenatal care clinic attendance register the Central Women’s Hospital. In general, a pregnant woman receives her antenatal care card after registration where basic information are recorded and updated at each visit. We selected women who were having their first pregnancy, had no known complications, had a single fetus, were 24 to 28 weeks of gestation, and lived in Yangon or nearby cities that had mobile phone network coverage. Other selection criteria were an absence of serious known illness that might potentially hinder breastfeeding, completed at least primary school education, and had access to a mobile phone. To explore the extent and nature of her husband’s support, gender roles and decision making power, we conducted in-depth interviews with accompanying husband. If husband was not available, mothers or mother-in-law were interviewed. For institutional staff, we included multiple stakeholders working in infant and young child feeding sector and purposively selected the respondents based on their expertise and areas of work.

We conducted ‘In –depth-interview’ (IDIs) with fourteen pregnant women, four husbands, one mother and one mother-in-law. One ‘Focus Group Discussion’ (FGD) was conducted with 8 nurses on duty (staff nurse, trained nurse and midwives), and KII with an obstetrician and a medical officer from the antenatal clinic at the hospital; program staff from National Nutrition Centre, Department of Health; Young Child Survival and Development Section, United Nations International Children’s Emergency Fund (UNICEF); Nutrition Section, Save the Children; Myanmar Maternal and Child Welfare Association (largest national NGO providing maternal and child health service,) and Ooredoo mobile company, one of the two recently launched mobile phone operators in Myanmar.

### Data collection

We used semi-structured in-depth interviews (IDI), key informant interviews (KII) and focus group discussions (FGD) to collect the data. Before starting the interviews, separate guidelines were developed for all IDIs and FGDs and later translated into Burmese language. In February, 2014, the interview guidelines were pretested (by MPH) with three pregnant women and three accompanying husbands at the study hospital. Brainstorming sessions with UNICEF and Save the Children staff were conducted and guidelines were revised accordingly based on their feedbacks. The data collection took place during June and July, 2014. With the help of the hospital staff and nurses, MPH filtered the potential respondents based on the hospital attendance registry book to assess the eligibility, approached the selected woman individually, explained the nature of study, provided the ‘information statement and consent form’ written in Burmese and obtained a signed consent form. If the respondent woman came with accompanying family member, MPH approached him or her and conducted an interview after receiving informed consent. We also obtained consent to record interviews with a digital recorder. The data was coded as numbers, reported as anonymous, participant’s confidentiality is assured and individual cannot be identified in reporting.

The major topics explored with the pregnant women and their family members included their socioeconomic background, perceptions and sources of information about infant feeding, women’s intention to breastfeed, and the expected barriers and enablers to practice exclusive breastfeeding. We also elicited women’s understanding on infant feeding practices among their friends and families. As the intended intervention uses mobile phone communications, we explored the women and their family’s access to mobile phones, their skills on using a mobile phone, their perceptions about the intervention, the preferred method of receiving the educational messages (Short Message Service (SMS) or direct phone calls) the timing and frequency of receiving the educational messages. The guidelines for KIs and FGDs with institutional health staff included views and suggestions on current infant and young child feeding practices, barriers for appropriate breastfeeding practices, and perceptions and recommendations on the planned intervention. Interviews with the mobile operator explored mobile network coverage plan, possible challenges and feasibility of implementing a mobile communications intervention and the potential to expand to rural populations in the future.

### Data analysis

All digitally recorded interviews were transcribed verbatim in Burmese by a local typist in Microsoft Word. A sample of transcript were as reviewed line by line by one of the authors (MPH) to check for accuracy and translated into English. These samples were then checked by AA for further quality check for the translation and interviewing process. Two of the authors (MPH and AA) discussed two initial transcripts to initiate the development of the codes. Subsequently all transcripts were carefully read by MPH to develop a list of thematic codes, which were independently reviewed and verified by AA. The data were manually coded by MPH for emerging themes, which was again verified by AA. Subsequently the text pertaining to each thematic code was discussed and summarised in a document that presents the findings for each theme using quotes and tables. Themes were triangulated using data collected through interviews and FGDs with different types of respondents.

## Results

### Demographic characteristics of the women

The pregnant women participated in this study were mostly in their mid-twenties. All of them were between 24 to 28 weeks of gestation, the majority were from Bamar followed by Chin, Kayin, and Indian and Chinese ethnicity. All women came from nuclear families and majority were Buddhist and lived in urban slum area. They had varying levels of education. Among 14 pregnant women, majority of them (42%, *n* = 6) completed middle school, one fifth (*n* = 3) completed primary school, 15% (*n* = 2) completed high school and 15% (*n* = 2) were graduate while a woman was a university student (distance learning). Among interviewee women, half of them were employed and working as vendors, casual workers or government staff.

### Perceptions of infant feeding and sources of information

Women and accompanying family members were aware of breast milk benefits for babies, colostrum and the correct time to initiate breastfeeding. Interestingly no woman mentioned the benefits of breast milk for themselves.

*“I know breast milk is good and has good elements.” (A pregnant woman, employed)*

*“I thought colostrum (noh-oo-ye) was same as breast milk, both come from breast, what is the difference?” (A grandmother, unemployed)*

Though most women had heard about exclusive breastfeeding for 6 months, detailed probing revealed that majority of them thought that exclusive breastfeeding meant giving breast milk only with no infant formula or other drinks or semi-solid foods, but small sips of water were acceptable. More than two thirds of women felt that breastfeeding alone for 6 months would not provide sufficient water and nutrients for their babies. Some women considered that mixed infant feeding (breast and infant formula) provided the best nutritional value.

*“I will practice exclusive breastfeeding and only give water via cotton bud to prevent dry mouth, hiccough, etc.” (A pregnant woman, employed)*

Majority reported that 2 to 4 months of age were the best timing to introduce complementary food. Regarding sources of information on infant feeding, respondents mentioned various sources, such as television, bill boards, pamphlets, health magazines, health staff, and family members especially mothers or mothers-in-law while no one mentioned of listening to radio as a source. A husband mentioned mobile internet as his source of knowledge. Although respondents acknowledged of hospital breastfeeding education activities, they claimed these were not enough.

*“Doctors examined only a few minutes, they are too busy and I scare to ask information to them.” (A pregnant woman, housewife)*

### Infant feeding practices in the community and influencing factors

All women noticed all mothers in their community fed breast milk to their babies while only one woman knew of a mother practiced exclusive breastfeeding for six months successfully. They felt that underlying socio-economic factors played an important role and work was the main reason for introducing infant formula. Two respondents from Ayeyarwady region (one of the most populous areas in Myanmar and occupy the delta region of the Ayeyarwady River) mentioned that in their villages, honey was given soon after delivery by traditional birth attendants due to cultural belief-for example- “the child needs honey to freshen up himself as he /she stays inside mothers’ womb for a long time”. Mixed feeding was the most commonly seen feeding pattern and water was found to be given as early as less than four weeks after delivery. Formula supplementation was commonly used before child reach 4 months and poor usually used formula made in China and the rich preferred branded infant formula such as Nestle or Dumex. A vast majority of respondents reported of knowing mothers introduced various kinds of soft and semi-solid foods at different age of children mostly at 2–4 months such as pasted rice powder or soft, mushy rice or chewed rice added by oil and salt. They noticed that mothers gave fruity ORS in hot weather, added blended carrots, beans or eggs while meat or dark green vegetables was not commonly given.

Perceived reasons for low exclusive breastfeeding rate in the community revealed as community did not believe that breast milk alone could provide all nutrients needed for children for 6 months, worried that child could be thirsty or hungry, beliefs that infant formula could enhance child memory or physical strength, work related constraints and grandmothers’ influences.

*“My niece is tall and smart. My sister fed her Dumex* (a branded infant formula)*, in addition to breast milk.” (A pregnant woman, employed)*

The findings from the nurses, doctors, program managers and staff working in the infant feeding promotion programs in Myanmar about the community current feeding patterns were similar to the participant women responses. Finding from the FGD with nurses and program staff revealed that mothers gave water followed by infant formula or condensed milk mixed with water even before they discharged from the hospital after delivery, mostly influenced by grandmothers.

*“After delivery, grandmothers or family members fed sips of water via cotton or spoon during hospital stay behind my back.” (A hospital nurse)*

Limited capacity to overcome barriers, accessibility to resources and poverty served as contributing factors for failed exclusive breastfeeding practices.

*“There are not many maternity leaves in private (industry) and no favorable breastfeeding environment, job security is more important for them.” (Nutrition program manager, non-governmental sector)*

### Intended feeding patterns and influencing factors for exclusive breastfeeding practice

Though all pregnant women intended to breastfeed, detailed probing revealed the women were prepared to add water, infant formula or other foods when their infants was from 1 to 4 months of age thus showing their determination was not as they stated. Women provided various examples as their intended feeding practices such as rice water, pasted rice powder, porridge, cooked or chewed rice when baby would be 2 to 4 months old, with added salt and oil. Upon exploring diversity of food to be added, carrots, peas, eggs and chicken were the most commonly examples given and they did not intend to add either ‘vegetables, meat or chicken or pork organs’ to prevent belching, hiccough, indigestion and worms*(than-hta).*

*“Will give rice meshed and sieved through a clean washed thin cloth at 2–4 months along with breastfeeding. Baby could be strong.” (A pregnant woman, employed)*

To practice exclusive breastfeeding successfully for six months was not an easy task for respondents and emerging barriers appeared as work as a major barrier for the working women, doubt on the quantity and quality of breast milk, grandmothers’ influences, limited ‘know-how ‘capacity and resources to overcome challenges . Some selected quotes were;

*“I need to get back to work, maternity leave is short, infant formula is an only option.” (A pregnant woman, employed)*

*“My mother said child will be small and short if we did not add other food.” (A pregnant woman, unemployed)*

Almost all women reported that they did not have any idea on how and where to access health information to overcome the barriers. Consequently, they reported low confidence level to manage breast problems if occurred.

*“How could I breastfeed if I have cracked nipples or mastitis, my sisters cried all the time when she had it.” (A pregnant woman, employed)*

Majority of them did not aware importance of position and attachment to have enough milk and techniques to express milk for working mothers*.*

*“I heard of express milk but never saw it. I don’t know how many hours I could keep it at room temperature or in the fridge.” (A pregnant woman, employed)*

In terms of supporting factors to exclusive breastfeeding, women mentioned about support from husband and mothers and mother-in-law. In all interviews, women said they had freedom of choice to breastfeed, however, couples reported the possibility of grandmothers of both sides’ influences. Not engaging in work outside home was also perceived as a supportive factor.

*“I will support my wife to breastfeed and will tell my mom* (living with them), *‘it is for your grandchild’” (A husband, employed).*

“*If my mom or mother-in-law insisted me to add infant formula or water, I need to follow.” (A pregnant woman, unemployed).*

### Respondent use of mobile phones

Most of the respondents had mobile phones and the rest shared phones with their husbands. All of their handsets were made in China and almost all of them installed Myanmar font in their handsets. At the time of the study, original setting of handsets available in Myanmar did not have Myanmar font installed in key pad and additional set up is needed and could be done in mobile shops. They mainly used mobile phones to communicate with their families and friends. A few of them preferred text messages to phone calls to save cost. None of them had heard or experienced of receiving health information via mobile phones. Husbands appeared to have better knowledge on using mobile phones than their wives and willing to share health promotion messages to their wives.

“*Our village recently has mobile network and my husband bought me this cheap mobile handset in previous month. I know how to make a call and answer phone.” (A pregnant woman, own small grocery store)*

*“I think it should not be a problem for me to access, read and understand incoming text messages.” (A pregnant woman, unemployed)*

### Opinions about a mobile phone based intervention

All women and their husbands, welcomed the idea of receiving health promotional SMS into their phones and had positive attitudes on program successes in receiving health education messages to their mobile phones did not appear to be a potential downside for respondents women, their husbands, mother and mother-in-law. They felt that sending SMS 2–3 times per week as appropriate frequency. No one described of feeling annoying by receiving SMS and some even reported that they were willing to spend money on mobile bill to get health information.

*“In receiving SMS, it will be good if there is a picture in SMS.” (A pregnant woman, unemployed)*

*“I will forget what I learnt form TV or radio, but for SMS, I can look at any time I want and I can keep at all time.” (A pregnant woman, employed)*

In examining service providers opinions on the planned mobile phone based intervention, there were generally positive views on the intervention idea. A few participants mentioned that the study would use the integrated approach by leveraging the power of mobile phones at perfect timing which could enhance the community mobilization to participate in the study. Findings from FGD with nurses reported that sending health promotion SMS could attract mothers than traditional distribution of pamphlets, however younger nurses had higher positive attitudes toward using mobile phone messages than older staff. All respondents agreed that using SMS as a service delivery would be the best modality for current moment and suggested to send SMS at evenings or weekends to avoid mothers’ busy time. Perceived barriers found to be the unreliable mobile networks, affordability for handset, time needed to spend to send SMS, recipients’ willingness to read SMS if there were too many messages, and possibility of changing phone numbers in six months’ time. A staff reported of a potential downside as participants did not receive SMS as claimed to be due to natural disaster or unforeseen circumstances.

Recommendations made were to explore the best timing to send SMS to the selected women; to use simple, easily understandable and culturally appropriate terms, to use relevant ethnic languages such as Shan, Karen or Kachin in some areas; to add interactive or pictorial messages for better attraction. Other suggestions came out as conducting a proper evaluation after intervention, arranging for more media coverage, sharing and advocating for political commitment in order to scaling up of similar design to the national level.

Some selected positive quotes were;

*“I prefer SMS, no not need special skill to use, not need to wait for airtime like Television or radio, can read anytime & could not be intervened by noises.” (Staff, Government sector)“The idea of using mobile phone as right time as country situations has been significantly changed and more political commitment to promote breastfeeding.” (Staff, non-government sector)*

In an in-depth interview with a staff from mobile operator revealed that, “*We are aiming to cover 95% of the country in coming 3 years.”* He supported the intervention idea in principle and reminded to work carefully in developing text messages in limiting numbers of characters (word count) to be put in a message and to consider and assess participants’ literacy level as reading text message need to have some level of education.

## Discussions

This study provides novel insight into community and service provider’s perspectives of a text messaging exclusive breastfeeding promoting intervention for pregnant women attending the largest public women hospital in Myanmar and highlights several aspects of contents and service delivery model that may affect users’ acceptance and impact of the intervention. The findings have significance for generating positive outcomes. Firstly, we could identify the gaps between pregnant women’s high awareness on the benefits of breastfeeding and potential low exclusive breastfeeding practice. Secondly, by analyzing users and service providers’ opinions on the planned intervention, we could integrate valuable information into the intervention design formulation. We do not have earlier similar Myanmar studies for comparisons as this study is, to our knowledge, the first study of its kind in Myanmar.

### Incorporating findings into intervention design

We incorporated study findings to review the design of the intervention and confirm inclusion criteria, service delivery model and SMS service contents development. Examples of SMS contents in relation to study findings were presented in the Table 1. Our qualitative study revealed that a substantial majority women came to the hospital after 28 weeks of gestation, findings were supported by clinic attendance records and nurses inputs. We, therefore, tailored inclusion criteria to 28–34 weeks of gestation and not limited to first time pregnancy only. Participating women’s educational status was also changed from ‘passed primary school’ to ‘able to read and write Myanmar language’ to ensure that we could have enough sample size when we recruit study population for the planned randomized controlled trial intervention. We also revised the design by recording two additional alternate phone numbers (husband or family members) to capture loss to follow up due to possibility of mobile numbers changed and added a training to recruiting women on how to use SMS with their own mobile handset.

**Table 1:** Examples of SMS content in relation to study findings

Topics	Examples SMS
Breast could quantity and quality	“Breast milk contains more than 80% water, it can satisfy your baby thirst, and you don’t need to give water till child is 6 months”. “Breast milk will give all the nutrients your baby need for healthy physical and brain development till 6 months.”
Colostrum’s benefit	“Colostrum (Noh-Oo-Ye) will protect your baby from allergy, infection and yellow skin and eye (A-Thar-Wah).”
Reducing pre-lacteal feeding	“Giving something else than breast milk will interfere (reduce) with breast milk production.”
Good for mothers (motivation)	“Breastfeeding will helps you reduce your weight after delivery. Your chance of having breast and ovarian cancer later in life will be reduced.”
Skilled-training approach	“Ensure that your baby’s tummy is touching your tummy, head and body is in line, whole body is supported, nose is facing the nipple and baby is able to look up at your face. CHEST TO CHEST; CHIN TO BREAST.”
Tips for managing breast problem	“If you have sore/cracked nipple or breast engorgement, gently message your breast.”
Tips for breast milk flow	“Frequent breast feeding can reduce pain & produce more breast milk.”
Working mothers	“Express milk can put safely in refrigerator for 72 h, at room temperature for 24 h and put in a clean and sealed container.”
Formula milk	“Formula milk may cause constipation because it is harder to digest than breast milk. Do not believe advertisement.”
Grandmothers	“Please show this message to grandmother: Do not give any fluid even water. Breast milk alone has all nutrients and water.”

Mobile phone, or use of SMS text messaging in particular, may provide an opportunity to improve health behaviors. According to the 2011 World Health Organization report, mobile phones provided a new communication channel for health promotion and community mobilization [13]. Several studies have shown feasible and promising results of using mobile phones and text messaging in smoking cessation [14–16], diabetes education [17], sexual health [18], diet and physical activity [19] as well as maternal and child health nutrition [20, 21]. Respondents preferences on the SMS over voice messages is consistent with study from Malawi [22] and we chose to use SMS message. As penetration of mobile phone has increased dramatically recently [23], unlike other countries, study participants were eager to receive health education messages to their mobile phones. In line with participants’ feedbacks, we created messages that were simple, locally acceptable, and culturally appropriate. For example, we included message as “Not to give honey after birth as breastmilk alone is the best for baby” to correctan incorrect belief commonly found in Ayeyarwady region. To be consistent with mobile operator‘s inputs, each message has approximately 160 characters written in Myanmar fonts (*Zawgyi font*^1^). Due to financial and technology constraints, we did not add pictorial images in the planned intervention. In response to respondents’ suggestions, we designed breastfeeding promotional messages to be delivered three times a week (Tuesday, Thursday and Saturday) to the intervention group and non breastfeeding related messages, mainly pregnancy and child care messages, to the control group once a week in the evening. We added two ways SMS communications model to monitor the delivery status of women. We hope that this formative study could contribute in promoting exclusive breastfeeding practices in Myanmar to some extent as promotion of nutrition drew a policy maker’s attention [24]. Perceived barriers provided by the participants and service providers such as mobile network coverage, electricity, mobile handset cost and community SMS familiarity were similar to other study findings [13, 22] and we considered these factors to design the intervention.

The limited understanding of the exact definition of exclusive breastfeeding might reflect the possible main reason for gap in high awareness on breastfeeding benefits and low exclusive breastfeeding practice. Respondents had inappropriate knowledge of nutrient contents in breast milk, misperception that water could be added in practicing exclusive breastfeeding, preferred formula milk or influence of formula milk advertisement. A study with 409 first-time Australian mothers from Western Sydney revealed similar finding. A limited understanding of the term exclusive breastfeeding was also reported in this study. A study in Brazil described that respondents had doubts on the quality of breast milk resulting in the feeding of water to a quarter of children in first month [25–28]. Unpublished Myanmar studies reported that estimated 70% of mothers gave water to their babies before one month. Reasons given for giving water in our study appeared as ‘water was natural, prevent thirst, dry mouth and thrush’ and similar to the findings from Nepal and Vietnam [29, 30]. Women expected their child to be tall, gain weight and be smart and adding formula milk or mixed feeding perceived as the best source for nutrients and convenient for weaning similar to the studies conducted in China, Bangladesh and other countries [31–33], leading to higher odd of failed exclusive breastfeeding for 6 months. We therefore incorporated correct information into the messages development. Example messages were “Breast milk will give all the nutrients your baby need for healthy physical and brain development” “Don’t believe formula milk’s advertisement. Breast milk is the best”. Respondents claimed various sources as information and as per finding from China study, they also claimed of “having limited communications with hospital staff” [31].

Expected barriers to practice exclusive breastfeeding revealed as need to return to work earlier than the baby is six months old, worry of having insufficient breast milk and breast problems, limited skills to solve constraints and grandmothers influences. In general, these findings were in line with other several studies [25, 26, 31, 33, 34]. A secondary data analysis on five East and South East Asia countries supported mothers’ employment as barrier to exclusive breastfeeding [35] and we planned to send frequent messages on how to express and store breastmilk and dangers of formula milk. To empower respondents in dealing with expected breast problems, we incorporated skilled trainings approach on how to do correct position and attachment, manage cracked and sore nipples. Though husband supported breastfeeding, mothers-in-law tended to be the main influential persons for adding water, honey and formula milk. Our findings confirmed previous research showing that grandmothers play a vital role in feeding practices [36, 37]. In contrast, interviews with grandmothers from Nepal reported that they would not give water or pre-lacteal foods [38]. Cultural influences somewhat existed [38] as Chinese and Indian background women reported more of mothers-in-law’s influence compared to other ethnic groups. According to the respondents, potential influencing grandmothers would be those living with them and they could be respondent women or husbands’ mothers. In consistent with a recommendation from a UK study, [36], we added special messages addressing grandmothers.

In developing SMS for intervention group, alongside the findings of this study, we reviewed and utilized the infant feeding literature including UNICEF and World Health Organization (WHO) breastfeeding guidelines [3, 39], Australia breast and infant feeding guidelines [40, 41], Ministry of Health, Uganda breastfeeding counselling messages [42]. Information Education and Communications (IEC) materials focusing on the infant feeding educational messages, produced by the Department of Health, Myanmar [43] and Save the Children, Myanmar [44] and a similar study conducted in China [21, 45, 46]. Developed messages were then translated into Burmese, pre-tested with 15 pregnant women visiting study setting prior to the actual intervention.

This study and the related intervention will be, to our knowledge, a first ever research in Myanmar on using mobile phone as a tool to promote exclusive breastfeeding practice. This paper attempts to provide important insight into the study women’s perceptions, practices and barriers to practice exclusive breastfeeding for six months and theirs opinions and attitudes towards mobile phone based intervention. By incorporating findings, this study leads to the development of an effective randomized controlled trial intervention using SMS as a communications tool and we aim to apply similar study design in other health education promotion interventions in future.

### Strengths and limitations

Our study has strengths as the researchers involved in whole process of developing guidelines, conducting interviews, transcribed verbatim and translating bilingual (English and Burmese), resulting in minimizing errors in translation and meaning. Secondly, the study includes views of women, their family members and stakeholders from the public, development and private sectors. The findings have evidence-based relevance for designing an intervention to improve exclusive breastfeeding in Myanmar. However, there are some issues that should be considered when interpreting the findings of this study. In this study, we did not aim to generalize the findings, rather we wanted to focus in prioritizing findings and feedbacks to inform the proposed intervention. The recommendations and findings were related to the planned intervention and has implications for that specific program. Future formative and or feasibility studies would be needed to inform the designs of intervention that targets any specific socioeconomic and ethnic groups in Myanmar or beyond.

## Conclusions

We incorporated findings to develop messages and determine the modality, inclusion criteria and tailored with gestation and child age, to be delivered in the randomized controlled trial intervention.

## Abbreviations

IDI, In-depth-interview; KII, Key informant interview; FGD, Focus Group Discussion; ANC, Antenatal care; NGO, Non-Government Organization; IYCF, Infant and Young Child Feeding; UNICEF, United Nations Children’s Fund; WHO, World Health Organization; EBF, Exclusive breastfeeding

## Footnotes

1 Myanmar font systems are not yet standardized and we selected the Zawgyi font, which is the most commonly used font in Myanmar.
